# The enhanced antitumor activity of bispecific antibody targeting PD-1/PD-L1 signaling

**DOI:** 10.1186/s12964-024-01562-5

**Published:** 2024-03-12

**Authors:** Tianye Li, Mengke Niu, Jianwei Zhou, Kongming Wu, Ming Yi

**Affiliations:** 1 Department of Gynecology, The Second Affiliated Hospital, Zhejiang University School of Medicine, Zhejiang University, Hangzhou, 310009 People’s Republic of China; 2 Zhejiang Provincial Clinical Research Center for Obstetrics and Gynecology, Hangzhou, China; 3 Cancer Center, Shanxi Bethune Hospital, Shanxi Academy of Medical Science, Tongji Shanxi Hospital, Third Hospital of Shanxi Medical University, Taiyuan, Shanxi People’s Republic of China; 4 Department of Breast Surgery, College of Medicine, The First Affiliated Hospital, Zhejiang University, Hangzhou, 310000 People’s Republic of China

**Keywords:** Cancer immunotherapy, Bispecific antibody, PD-L1, PD-1, CD47, VEGF, 4-1BB, TGFβ

## Abstract

The programmed cell death 1 (PD-1) signaling pathway, a key player in immune checkpoint regulation, has become a focal point in cancer immunotherapy. In the context of cancer, upregulated PD-L1 on tumor cells can result in T cell exhaustion and immune evasion, fostering tumor progression. The advent of PD-1/PD-L1 inhibitor has demonstrated clinical success by unleashing T cells from exhaustion. Nevertheless, challenges such as resistance and adverse effects have spurred the exploration of innovative strategies, with bispecific antibodies (BsAbs) emerging as a promising frontier. BsAbs offer a multifaceted approach to cancer immunotherapy by simultaneously targeting PD-L1 and other immune regulatory molecules. We focus on recent advancements in PD-1/PD-L1 therapy with a particular emphasis on the development and potential of BsAbs, especially in the context of solid tumors. Various BsAb products targeting PD-1 signaling are discussed, highlighting their unique mechanisms of action and therapeutic potential. Noteworthy examples include anti-TGFβ × PD-L1, anti-CD47 × PD-L1, anti-VEGF × PD-L1, anti-4-1BB × PD-L1, anti-LAG-3 × PD-L1, and anti-PD-1 × CTLA-4 BsAbs. Besides, we summarize ongoing clinical studies evaluating the efficacy and safety of these innovative BsAb agents. By unraveling the intricacies of the tumor microenvironment and harnessing the synergistic effects of anti-PD-1/PD-L1 BsAbs, there exists the potential to elevate the precision and efficacy of cancer immunotherapy, ultimately enabling the development of personalized treatment strategies tailored to individual patient profiles.

## Background

Programmed cell death 1 (PD-1) signaling acts as a fundamental immune checkpoint mechanism, downregulating inflammatory responses and maintaining immune homeostasis [1]. Key structures within PD-1, namely the immune receptor tyrosine-based inhibitory/switch motif (ITIM/ITSM), facilitate signal transduction and recruit phosphatases (SHP1/2) within the cell [2]. The PD-1/PD-L1 signaling not only serves as a crucial pathway for preventing autoimmune diseases, but also significantly influences the delicate balance between tumor immune surveillance and immune tolerance [3]. Increased PD-L1 on tumor cells or infiltrating lymphocytes can result in T cell exhaustion, dampening tumor-specific immunity and promoting tumor progression [4]. PD-1/PD-L1 inhibitors have emerged as a groundbreaking therapeutic approach by blocking the negative regulatory signals, effectively releasing T cells from their exhausted state [5]. Since the approval of the first anti-PD-1 antibody (pembrolizumab) by the FDA in 2014, PD-1/PD-L1 blockade therapies have revolutionized clinical practice, exhibiting potent and durable antitumor effects, particularly in refractory tumors [5–10].

PD-1/PD-L1 inhibitors function by disrupting the immunosuppressive signals that tumors exploit, allowing immune cells to recognize and kill cancer cells more effectively [11]. The clinical successes of anti-PD-1/PD-L1 agents have underscored the importance of immune checkpoint blockade in cancer therapeutics [12–17]. However, challenges such as resistance, limited response rates, and adverse effects have prompted the exploration of innovative strategies to optimize and broaden the therapeutic impact [18, 19]. In tandem with these developments, bispecific antibodies have emerged as a promising frontier in cancer immunotherapy [20]. By simultaneously targeting PD-L1 and other key molecules involved in immune regulation, bispecific antibodies (BsAbs) offer a multifaceted approach to enhance antitumor immune responses [21–23]. This review delves into the recent advancements in PD-1/PD-L1 blockade and explores the potential of bispecific antibodies, with a focus on their development and application in solid tumors. By elucidating the advances in anti-PD-L1 BsAb development, especially those tailored for solid tumors, this review aims to contribute to the evolving understanding of cancer immunotherapy and pave the way for more effective and personalized treatment strategies.

## The advances of BsAb

### The development of BsAb

**Fig. 1:**
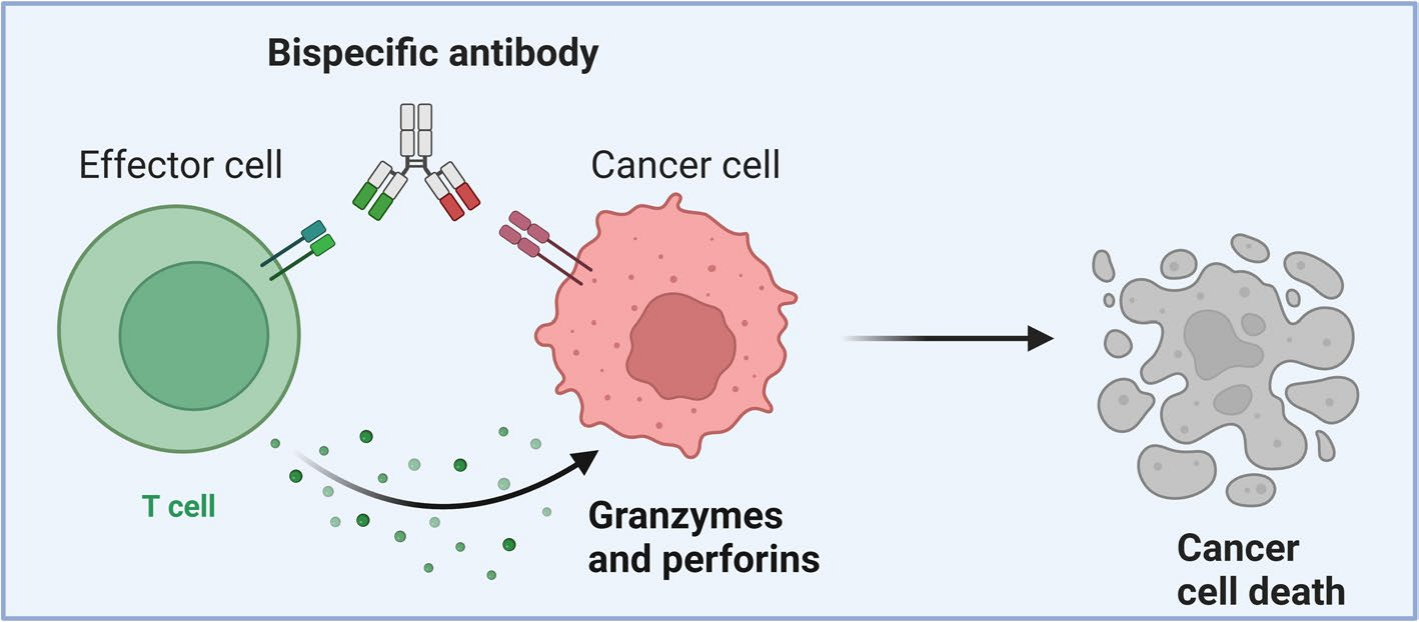
Bispecific antibodies (BsAbs) enhance tumor killing by guiding various effector cells to tumor cells in a non-MHC-restricted manner. BsAbs facilitate the interaction between T cells and tumor cells, triggering a sequence of events leading to T cell activation. The primary mechanism employed by activated T cells in cancer cell lysis involves Granzyme-B and perforin (Adapted from “Bispecific Antibody Mechanism of Action”, by BioRender 2023)

**Fig. 2:**
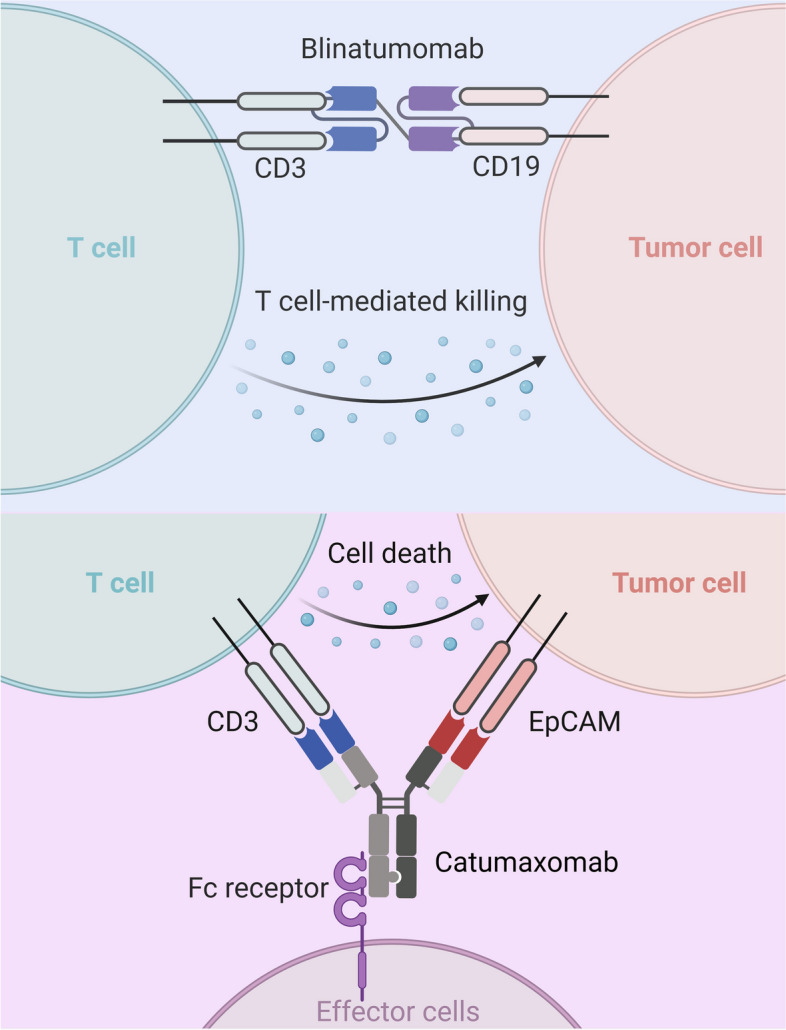
The tumor-killing mechanisms of blinatumomab and TrioMabs. Blinatumomab is an anti-CD3 × CD19 bispecific T-cell engager (BiTE) antibody. Blinatumomab is designed to bind to both CD19 of B cells and CD3 of T cells. By linking these two cell types, blinatumomab helps facilitate the T cell response against cancer cells, leading to the destruction of B-cell leukemia cells. Catumaxomab is an anti-CD3 × EpCAM BsAb based on TrioMabs technique, binding to EpCAM of cancer cells and CD3 of T cells. Notably, the Fc domain could bind to Fcγ receptor of effector cells including NK cells, macrophages, and dendritic cells, triggering antibody-dependent cell cytotoxicity or phagocytosis, and complement-dependent cytotoxicity against cancer cells (Adapted from “Bispecific Antibody Design”, by BioRender 2023)

The success of monoclonal antibodies targeting tumor-associated antigens (TAAs), such as Her2 or EGFR, in breast and lung cancer therapy has led to the exploration of innovative approaches, including the development of BsAbs [24]. BsAbs, introduced in the 1980s, have garnered considerable attention for their potential in cancer treatment [25]. Functionally, BsAbs serve as effective linkages between immune effector cells and tumor cells, or concurrently block two different oncogenic molecules [26]. Besides, some BsAbs enhance tumor killing by guiding various effector cells to tumor cells in a non-MHC-restricted manner (Fig. 1) [26]. Advancements in technology have resulted in various BsAb formats, classified based on the Fc domain into non-IgG-format and IgG-format. IgG-like agents retain Fc-mediated antibody effector functions, while Fc-free BsAbs lack these functions [26]. Bispecific T cell engagers (BiTEs) and Triomabs are prominent BsAb formats [27, 28]. BiTEs, lacking Fc domains, exhibit short serum half-lives, limiting their clinical application [28, 29]. Triomabs, with an IgG-like structure, show slower clearance but face challenges of immunogenicity and compromised permeability due to the Fc domain (Fig. 2) [30].

In the last decade, the development of bispecific antibodies has been dominated by BiTEs. These antibodies, which simultaneously bind CD3 of T cells and TAAs of tumor cells, activate T-cell signaling cascades and initiate target-dependent tumor cell killing [26]. Unlike checkpoint inhibitors, BiTEs overcome major histocompatibility complex (MHC) restrictions of the T-cell receptor (TCR), presenting a breakthrough validated in the clinic with FDA approvals for blinatumomab (anti-CD3 × CD19) [31–33]. Besides, the anti-CD3 × CD20 BsAb mosunetuzumab has been approved for refractory or relapsed follicular lymphoma as well [34, 35]. However, despite the promising outcomes observed in hematological malignancies, the therapeutic effects of bispecific antibodies in solid tumors, which constitute 90% of all cancers, remain a challenge, primarily due to the suppressive tumor microenvironment (TME) impairing T-cell activity and fostering immune deficiency [33, 36–38].

**Fig. 3:**
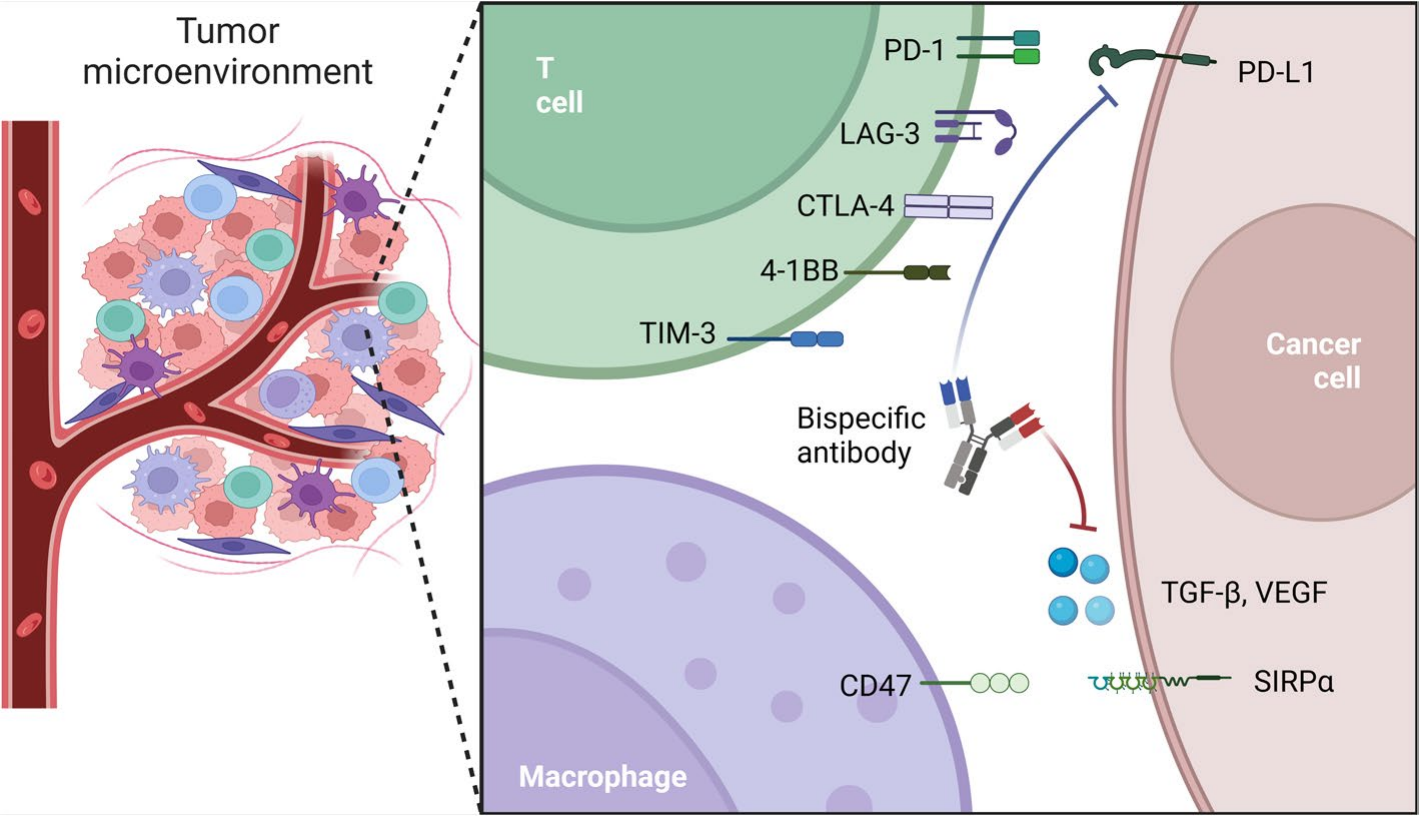
BsAbs simultaneously targeting two immunoinhibitory molecules on tumor cells or cytokines in the TME. In contrast to BiTEs, these bispecific antibodies aim to block two immunoinhibitory signaling pathways (except 4-1BB agonist antibodies), generating synergistic anti-cancer effects or minimizing drug resistance (Created with Biorender)

Another avenue of BsAb investigation involves simultaneously targeting two epitopes on tumor cells or cytokines in the TME (Fig. 3). In contrast to BiTEs, these bispecific antibodies aim to block two protumor signaling pathways, generating synergistic anti-cancer effects or minimizing drug resistance [39]. For instance, bifunctional antibody M7824, targeting PD-L1 and TGFβ, has exhibited significant clinical efficacy in non-small cell lung cancer (NSCLC) patients [40]. Besides, Although BsAb clinical outcomes are less satisfying in solid tumors compared to hematologic malignancies, ongoing studies and clinical trials, particularly focusing on commonly expressed antigens (*e.g.* EpCAM, HER2, PSMA, and CEA), demonstrate the great potential of BsAb in cancer immunotherapy [41]. Recently, BsAbs simultaneously targeting PD-L1 and other immunoinhibitory molecules have been developed. These BsAbs show potent antitumor activity in preclinical and clinical studies, regarded as the next generation of immune checkpoint inhibitors (ICIs) [42–44].

### The challenges for BsAb in solid tumors

In addressing solid tumor malignancies, BsAbs encounter significant hurdles that impede their clinical success. Predominantly, these challenges include managing adverse reactions associated with treatment, mitigating both on-target and off-target toxicities, and navigating the intricacies of the immunosuppressive TME [41]. A critical issue associated with BsAbs, especially those with intact Fc domains, is the risk of off-target toxicity, exemplified by Cytokine Release Syndrome (CRS) [45]. CRS is a systemic inflammatory reaction characterized by a spectrum of clinical manifestations, from mild symptoms to severe, potentially fatal conditions, often marked by laboratory signs such as cytopenia [46]. The pathophysiology of CRS involves an immune cascade triggered by IFN-γ release from activated T cells, which subsequently prompts macrophages to produce an excess of inflammatory cytokines [41]. A significant contributor to this issue is the inadvertent T-cell activation, which can occur through mechanisms like FcγR binding on non-target cells [47].

The standard mitigation strategy involves corticosteroid pretreatment and optimized dosing [48]. Furthermore, targeting IL-6, a key cytokine in CRS pathogenesis, with antagonists like tocilizumab has shown promise in alleviating these adverse effects without compromising the antitumor efficacy of BsAb therapies [49]. Besides, innovations in BsAb design, like employing Fc-free formats or antibodies with modified Fc domains, are crucial to reduce these risks [50]. Besides, on-target toxicity links directly to the target specificity of BsAbs. While certain tumor-associated antigens (TAAs) demonstrate suitability, others pose risks due to their presence in normal tissues, leading to significant toxicity. For example, BsAbs targeting EpCAM have shown this problem [51]. Moreover, the strong affinity of BsAbs to their targets can lead to on-target CRS. However, unlike tissue toxicity, on-target CRS is typically transient and can be managed with dose modulation and supportive care [47].

Besides, the effectiveness of BsAbs in solid tumors is critically impacted by the TME. A significant challenge is the insufficient T-cell infiltration in immune-desert tumors, which limits BsAb efficacy [52]. Innovative interventions, such as the use of oncolytic reovirus, have been employed to improve T-cell infiltration, transforming 'cold' tumors into more responsive 'inflamed' ones, thereby overcoming resistance to the T cell-engaging BsAb therapy [53]. Additionally, the immunosuppressive nature of the TME, marked by the upregulation of PD-1 and PD-L1 during BsAb therapy, presents another hurdle [54]. The moderate efficacy of BiTEs in solid tumors has led to the exploration of adjunct therapies like checkpoint inhibitors and T-cell costimuli to enhance their antitumor activity. Such combinations have shown promise in preclinical studies by overcoming T-cell exhaustion and amplifying BiTE effectiveness [55, 56]. Additionally, strategies like combining BiTEs with T-cell costimuli like 4-1BB agonists, have been effective in boosting BiTE performance [57]. In sum, combining BsAbs with other immunotherapies can enhance therapeutic efficacy, though results vary depending on BsAb composition, target antigen, and tumor types. Thus, the complex dynamics between BsAbs and the TME in solid tumors require multifaceted and innovative therapeutic strategies to fully harness their potential.

## Anti-TGFβ × PD-L1 BsAb

### The role of TGFβ in cancer immunology and immunotherapy

The transforming growth factor beta (TGFβ) signaling pathway exhibits a dual nature in cancer biology, serving both tumor-suppressing and tumor-promoting roles, depending on the specific cell and tissue context [58]. In normal cells, TGFβ functions to maintain cellular homeostasis and prevent tumor initiation, primarily by arresting the cell cycle, promoting cellular differentiation, and triggering cell apoptosis [59]. The pathway's response varies across cell types due to differential expression of factors like Smad proteins [60]. Contrastingly, in cancer cells, the regulatory role of TGFβ is often disrupted or altered due to mutations or epigenetic changes, leading to a shift from controlling proliferation to facilitating cancer progression [61]. In the TME, hyperactive TGFβ signaling, typically inhibitory in normal epithelial cells, paradoxically supports tumor growth, invasion, and metastatic behavior [62]. Notably, TGF-β-induced epithelial to mesenchymal transition (EMT) is crucial in cancer development, invasion, and spread [63, 64]. The flexibility and reversibility of EMT in response to TGFβ levels underscore its significance as a potential therapeutic target, especially since it fosters a stem-like phenotype linked to tumor progression and resistance to chemotherapy [65, 66]. A comprehensive understanding of TGFβ's contrasting roles in different cancer types and tissues, along with its impact on the TME, is essential for devising targeted treatments to curb cancer progression.

Notably, accumulating evidences demonstrate that TGFβ stands as a central player in the intricate landscape of cancer immunology and immunotherapy, exerting dual effects on tumorigenesis and immune modulation. Its role in the TME is multifaceted, as it not only contributes to the promotion of tumorigenesis but also establishes an immunosuppressive milieu that shields cancer cells from immune surveillance [44]. The immunosuppressive functions of TGFβ are manifested through its ability to inhibit the activation and function of various immune cells, including NK cells, T cells, and dendritic cells (DCs) [67–69]. Moreover, TGFβ enhances the differentiation and expansion of immunosuppressive regulatory T cells (Tregs), further tilting the balance in favor of immune evasion by cancer cells [70]. In the context of cancer immunotherapy, the immunosuppressive nature of TGFβ poses a significant hurdle. Strategies aimed at neutralizing or inhibiting TGFβ signaling have emerged as promising avenues to enhance the efficacy of immunotherapies [71]. Notably, the development of anti-TGFβ × PD-L1 BsAb represents a groundbreaking approach to simultaneously target multiple immunosuppressive pathways within the TME, thereby unleashing the full potential of the immune system against cancer.

It is noteworthy that the dual role of TGFβ in cancer underscores the necessity of understanding its contextual influences for effective patient selection in anti-TGFβ therapies. The pleiotropic activities of TGFβ signaling pose a challenge in developing antagonists for cancer treatment, particularly due to the lack of specific biomarkers and established dosing regimens [60]. To integrate TGFβ blockade agents effectively into frontline cancer therapy, future clinical trials need to focus on bioinformatics and identifying molecular biomarkers for patient stratification and treatment optimization.

### M7824 and other bifunctional antibodies

M7824, a novel bifunctional fusion protein, represents a significant stride in PD-L1 × TGFβ dual-blockade therapy (Table 1). This innovative agent combines an anti-PD-L1 domain in the Fab with a TGFβ receptor in the Fc, allowing for simultaneous targeting of both immunosuppressive pathways. M7824 was designed to target PD-L1 molecules on tumor cell, localizing a trap molecule in the TME to capture immunosuppressive TGF-β. Then, M7824 is internalized by cells expressing PD-L1, leading to the removal of M7824-bound TGF-β [42]. In theory, M7824 is expected to exhibit greater specificity for tumor cells compared to a combination of two monoclonal antibodies due to its physical bridging effect. In animal models, M7824 exhibited potent antitumor efficacy, obviously retarding the tumor growth and prolonging survival [42]. Beyond its direct antitumor effects, M7824 induced a substantial reshaping of the TME, including the prevention or reversal of TGFβ-mediated epithelial-mesenchymal transition in cancer cells [72]. This alteration enhances tumor cell susceptibility to immune-mediated attack and chemotherapeutic agents. The fusion protein upregulated the quantities and activities of cytotoxic lymphocytes while concurrently decreasing the proportions of immunosuppressive subsets, including Tregs, myeloid-derived suppressor cells (MDSC), and M2-like macrophages [42]. Additionally, M7824 induced tumor matrix remodeling, contributing to improved immune cell infiltration and reinforcing its potential as a multifaceted immunotherapeutic agent [42]. Moreover, when combined with radiation, chemotherapy, and other immunotherapeutic agents, it enhances overall antitumor activity [73]. In the phase 1 trial, M7824 provided promising responses, particularly in NSCLC with high PD-L1 expression (NCT02517398) (Table 2) [40, 43].

**Table 1:** The advances of bispecific/bifunctional antibodies targeting PD-1/PD-L1 signaling

Targets	Agents	Maximum R&D stage	Original Drug Company/Authors
TGFβ × PD-L1	M7824 (Bintrafusp alfa)	Phase 3	Merck KGaA
	YM101/BiTP	Phase 1/2	Wuhan YZY Biopharma
	SHR-1701	Phase 3	Hengrui Pharmaceuticals
	BR102	Phase 1	BioRay Pharmaceutical
	TQB-2858	Phase 2	Nanjing Shunxin Pharmaceutical
	PM8001	Phase 1/2	Pumis Biotechnology
	TST005	Phase 1	Suzhou Transcenta Therapeutics
TGFβ × PD-1	JS201	Phase 1/2	Junshi Biosciences
CD47 × PD-L1	IBI322	Phase 2	Innovent Biologics
	PF-07257876	Phase 1	Pfizer Inc
	6MW3211	Phase 2	Mabwell Bioscience
	IMM2520	Phase 1	ImmuneOnco Biotechnology
	BAT-7104	Phase 1	Bio-Thera Solutions
	IBC0966	Phase 1/2	Beijing Sunho Pharmaceutical
VEGF × PD-1	AK112 (Ivonescimab)	Application for market	Akeso Biopharma
VEGF × PD-L1	PM8002	Phase 2/3	Pumis Biotechnology
	B1962	Phase 1	Shanghai Tasly Pharmaceutical
	HB0025	Phase 2	Zhejiang Huahai Pharmaceutical
4-1BB × PD-L1	ABL503	Phase 1	ABL Bio
	PM1003	Phase 1/2	Pumis Biotechnology
	PRS-344/S095012	Phase 1/2	Servier Bio-Innovation LLC
	HK010	Phase 1	HankeMab
	GEN1046	Phase 1	Genmab
LAG-3 × PD-L1	IBI323	Phase 2	Innovent Biologics
	ABL501	Phase 1	ABL Bio
	FS118	Phase 1/2	InvoX Pharma Limited
LAG-3 × PD-1	INCA32459	Phase 1	Incyte Corporation
	Tebotelimab (MGD013)	Phase 3	MacroGenics
	RO7247669 (Tobemstomig)	Phase 2	Hoffmann-La Roche
	EMB-02	Phase 1/2	Shanghai EpimAb Biotherapeutics
CTLA-4 × PD-1	QL1706	Phase 2/3	Qilu Pharmaceutical
	MGD019 (Lorigerlimab)	Phase 2	MacroGenics, Inc
	XmAb2071 (Vudalimab)	Phase 2	Xencor
	AK104 (Cadonilimab)	Approved listing (Cervical cancer)	Akeso Biopharma
	MEDI5752	Phase 1	AstraZeneca PLC
CTLA-4 × PD-L1	KN046	Phase 3	Jiangsu Alphamab Biopharmaceuticals
TIM-3 × PD-L1	LY3415244	Phase 1	Eli Lilly
TIM-3 × PD-1	AZD7789	Phase 2	AstraZeneca PLC
	RO7121661 (Lomvastomig)	Phase 2	Hoffmann-La Roche
	LB1410	Phase 1	L & L biopharma
PD-1 × PD-L1	IBI318	Phase 2	Innovent Biologics
	LY3434172	Phase 1	Eli Lilly
TIGIT × PD-1	AZD2936 (Rilvegostomig)	Phase 3	AstraZeneca PLC
	IBI321	Phase 1	Innovent Biologics
TIGIT × PD-L1	HLX301	Phase 2	Shanghai Henlius Biopharmaceuticals
CD27 × PD-L1	CDX-527	Phase 1	Celldex Therapeutics

**Table 2:** The results of representative clinical trials of bispecific/bifunctional antibodies targeting PD-1/PD-L1 signaling

Targets	Agents	Clinical trials	Cancer types	Results
TGFβ × PD-L1	M7824	NCT02517398	NSCLC	ORR of PD-L1-positive at 1200 mg dose: 36.0%; ORR of PD-L1-high at 1200 mg dose: 85.7%
	SHR-1701	NCT05179239	Cervical cancer	ORR: 15.6%; DCR: 50.0%
		NCT03710265	Solid tumors	Clinical-expansion part of gastric cancer cohort: ORR of 20.0% and 12-month OS rate of 54.5%
	TQB2858	CTR20213001	Osteosarcoma and ASPS	PFS: 8.34 months; OS: 9.63 months
	PM8001	ChiCTR2000033828	Advanced solid tumors	RP2D: 20 mg/kg Q2W; ORR: 10.4%; DCR: 53.7%
	TST005	NCT04958434	Advanced solid tumors	DCR: 63.6% (SD, none achieving CR or PR)
CD47 × PD-L1	PF-07257876	NCT04881045	Advanced solid tumors	DoR: 16 weeks for SCCHN; ORR: 5.6%
VEGF × PD-1	AK112 (Ivonescimab)	NCT04736823	NSCLC	SCC: ORR: 75%, median DOR: 15.4 months, DCR: 95%; non-SCC: ORR: 55%, DCR: 100%
		NCT04900363	NSCLC	For all patients: ORR: 39.8%, DCR: 86.1%; ORR (TPS < 1%): 14.7%, ORR (TPS ≥ 1%): 51.4%, ORR (TPS ≥ 50%): 57.1%. ORR of dose at 30 mg/kg Q3W for PD-L1 positive: 75.0%
VEGF × PD-L1	HB0025	NCT04678908	Advanced solid tumors	ORR of dosed at ≥ 3 mg/kg Q2W: 9.1%; DCR of dosed at ≥ 3 mg/kg Q2W: 50%
	PM8002	ChiCTR2200060400	TNBC	ORR: 69.2%; DCR: 92.3%
		ChiCTR2000040552	Advanced solid tumors	ORR: 15.2%; DCR: 75.4%. ORR (cervical cancer):28%; ORR (renal cell carcinoma): 26.9%; ORR (platinum-resistant ovarian cancer): 15.4%; ORR (NSCLC with EGFR mutations): 18.5%
		ChiCTR2200059911	SCLC	ORR: 72.7%; DCR: 81.8% (18/22); Median PFS: 5.5 months
4-1BB × PD-L1	PM1003	ChiCTR2100052887	Advanced solid tumors	ORR: 5.6%; DCR: 44.4%
	GEN1046	NCT03917381	Advanced solid tumors	DCR: 65.6%
LAG-3 × PD-L1	FS118	NCT03440437	Advanced solid tumors	RP2D: 10 mg/kg weekly; DCR: 46.5%
LAG-3 × PD-1	Tebotelimab (MGD013)	NCT04178460	Gastric cancer	RP2D: 600 mg Q2W; ORR: 5.3%; DCR: 52.6%; Median PFS: 2.7 months; Median OS: 6.5 months
		NCT03219268	Advanced solid tumors	RP2D: 600 mg Q3W; ORR: 19%
	EMB-02	NCT04618393	Advanced solid tumors	DCR: 44.7%; CBR-24 (CR + PR + durable SD [≥ 24weeks]): 19%
	RO7247669	NCT04140500	Advanced solid tumors	ORR: 17.1%; DCR: 51.4%
CTLA-4 × PD-1	QL1706	NCT05329025	NSCLC	Wild-type EGFR: ORR: 45%; median PFS: 6.8 months; Mutated EGFR: ORR: 54.8%; median PFS: 8.5 months
		NCT05603039	Advanced HCC	ORR: 38.3%; DCR: 74.5%; Median PFS: 6.7 months
		NCT04296994 and NCT05171790	Advanced solid tumors	RP2D: 5 mg/kg; ORR: 16.9%, 14.0% (NSCLC), 24.5% (NPC), 27.3% (cervical cancer), 7.4% (colorectal cancer), 23.1% (SCLC); DoR: 11.7 months
		NCT05179317	Cervical cancer	ORR: 81%; DCR: 98.3%; Median PFS: 14.3 months
		NCT05309629	ES-SCLC	ORR: 89.7%; DCR: 97.4%
	MGD019 (Lorigerlimab)	NCT03761017	Advanced solid tumors	ORR: 16%; DCR: 36%
	AK104 (Cadonilimab)	NCT05522894	ESCC	ORR1: 86.7%; DCR: 100.0%; ORR (PD-L1 CPS ≥ 10): 83.3%; ORR (PD-L1 CPS < 10): 88.9%
		NCT03852251	Advanced solid tumors	ORR (Cervical cancer): 32.3%; ORR (ESCC): 18.2%; ORR (HCC): 19.6%
		NCT04646330	NSCLC	ORR of dosed at 15 mg/kg Q3W: 51%; ORR of dosed at 10 mg/kg Q3W: 60%
		NCT04444167	HCC	Dosed at 6 mg/kg Q2W: ORR: 35.5%; Median DoR: 13.6 months; Median PFS: 8.6; Median OS was 27.1 months; Dosed at 15 mg/kg Q3W: ORR: 35.7%; DCR: 13.67 months; Median PFS: 9.8 months
CTLA-4 × PD-L1	KN046	NCT03872791	TNBC	ORR: 44.0%; PFS: 7.33 months; OS: 30.92 months; PD-L1 + PFS: 8.61 months; 2-year OS rate: 62.5%; PD-L1-: PFS: 4.73 months; 2-year OS rate: 57.1%
		NCT03838848	NSCLC	Dosed at 3 mg/kg Q2W: ORR: 13.3%; PFS: 3.68 months; OS: 19.7 months Dosed at 5 mg/kg Q2WW: ORR: 14.7%; PFS: 3.68 months; OS: 13.04 months
		NCT04469725	Thymic carcinoma	ORR: 16.3%; DoR: 10.1 months; PFS: 5.6 months; 18-months OS rate: 74.1%
		NCT04521179	HER2 + gastric or GEJ cancer	ORR: 77.8%; DCR: 92.6%
		NCT04324307	PDAC	ORR: 11.1%; DCR: 44.4%; PFS: 2.1 months; OS: 7.5 months
		NCT03925870	ESCC	ORR: 58.3%; DCR: 91.6%
TIM-3 × PD-L1	LY3415244	NCT03752177	Advanced solid tumors	1/12 reaching PR
TIM-3 × PD-1	AZD7789	NCT04931654	Advanced NSCLC	7/19 reaching SD
PD-1 × PD-L1	IBI318	NCT03875157	Advanced solid tumors	3/9 of dose ≥ 10mg Q2W reaching PR
TIGIT × PD-1	AZD2936 (Rilvegostomig)	NCT04995523	NSCLC	ORR: 3.9%; DCR: 43.4%

Annotations: *ORR* Objective response rate, *DCR* Disease control rate, *OS* Overall survival, *NSCLC* Non-small cell lung cancer, *ASPS* Alveolar soft part sarcoma, *PFS* Progression free survival, *CR* Complete response, *PR* Partial response, *SCCHN* Squamous cell carcinoma of the head and neck, *DoR* Duration of response, *TPS* Tumor proportion score, *TNBC* Triple-negative breast cancer, *RP2D* Recommended phase 2 dose, *SD* Stable disease, *HCC* Hepatocellular carcinoma, *NPC* Nasopharyngeal carcinoma, *SCLC* Small cell lung cancer, *ESCC* Esophageal squamous cell carcinoma, *GEJ* Gastroesophageal junction, *PDAC* Pancreatic ductal adenocarcinoma

The success of M7824 has catalyzed the exploration and development of additional anti-TGFβ × PD-L1 bifunctional proteins landscape. Among these, SHR-1701, with a structure reminiscent of M7824, combines anti-PD-L1 domain with an N-terminal-truncated TGFβRII [74]. In a phase 1 clinical trial (NCT05179239), SHR-1701 exhibited antitumor activity in recurrent metastatic cervical cancer [75]. Similarly, the bifunctional protein BR102, comprising an anti-PD-L1 antibody and TGFβRII ectodomain, demonstrated antitumor activity in murine tumor models [76]. These emerging antibodies, including SHR-1701 and BR102, contribute to the expanding repertoire of potential anti-TGFβ × PD-L1 blockade therapies, promising novel therapeutic strategies for the complex landscape of cancer immunotherapy.

### YM101 and BiTP

YM101, heralded as the world's first publicly reported anti-TGFβ × PD-L1 BsAb, marks a pivotal advancement in the field of cancer immunotherapy. Engineered using the Check-BODY™ technology platform, YM101 represents a testament to the innovative strategies employed to combat the dual challenges posed by PD-L1 and TGF-β [21]. Preclinical investigations revealed YM101's ability to effectively counteract the effects of both TGF-β and PD-1 × PD-L1 signaling. Moreover, in vivo evidence demonstrated that YM101 outperformed anti-TGF-β and anti-PD-L1 monotherapies in terms of antitumor activity. We hypothesize that this improved antitumor effect may be attributed to the enhanced tumor specificity resulting from the distinctive physical bridging effect of YM101. However, it is crucial to acknowledge that our current speculation lacks experiment evidence to substantiate it. In upcoming research, it will be imperative to employ techniques such as isotope labeling to further validate and demonstrate the advantages of YM101, specifically in terms of its potential for increased tumor specificity and the associated enhancement of antitumor effects.

Besides, YM101 played a transformative role in shaping the TME, promoting the formation of inflamed tumors characterized by increased numbers and activities of tumor-infiltrating lymphocytes (TIL) [21]. Additionally, YM101 shifted the balance of macrophage polarization towards the antitumor M1 phenotype, further enhancing its immunotherapeutic potential [21]. Besides, in preclinical studies, the combination of STING agonists and YM101 demonstrated potent and durable antitumor immune protection by targeting three independent and complementary pathways [77]. STING agonists induce DC maturation and activate macrophages, reigniting immunologically cold tumors and enhancing both innate and adaptive immune responses systemically. When combined with YM101, STING agonists synergized to normalize the TME and impede tumor growth in non-inflamed models [78].

Inspired by the encouraging preclinical results, the development of the alternative molecule for clinical trials (BiTP) followed suit. Sharing a similar structure with YM101 and constructed using the Check-BODY™ platform, BiTP demonstrated efficacy in murine triple-negative breast cancer (TNBC) models [79]. Efficacy experiments in humanized TNBC models indicated that BiTP exhibited superior antitumor efficacy compared to corresponding monotherapies. BiTP reduces collagen generation, enhances T-cell penetration, and increases the infiltration of lymphocytes into the tumor [79]. At the present stage, multiple clinical trials of BiTP are ongoing, including CTR20211776 (for solid tumors) and CTR20223410 (for pancreatic cancer). Generally, the development of anti-TGF-β × PD-L1 BsAb, exemplified by YM101, BiTP, and M7824, represents a transformative approach to cancer immunotherapy [61, 80, 81]. These innovative agents, designed to concurrently target multiple immunosuppressive pathways, have shown remarkable efficacy in preclinical and clinical settings. The synergistic effects observed in combination therapies further underscore the potential of these antibodies to overcome resistance mechanisms and broaden their applicability across diverse tumor types.

## Anti-CD47 × PD-L1 BsAb

CD47 plays a pivotal role in cancer by delivering a "don't eat me" signal to macrophages when binding to its ligand signal-regulatory protein alpha (SIRPα) on tumor cells [82]. Antibodies disrupting CD47 or its ligand have shown therapeutic effects in preclinical studies and clinical trials [83]. CD47 blockade enhances antigen presentation, phagocytosis, and immune infiltration in various tumor models, supporting the development of CD47 blockade immunotherapy agents [84–87]. Furthermore, the dual blockade of CD47/SIRPα and PD-1/PD-L1 signaling, which respectively suppress innate and adaptive immune responses, has shown enhanced therapeutic efficacy in various cancer types, providing a promising avenue for cancer treatment that stimulates both arms of the immune system [88]. Based on knobs-into-holes (KIH) platform, Wang et al*.* developed an anti-CD47 × PD-L1 BsAb 6MW3211, which was designed with a common light chain, exhibiting low affinity to CD47 and high affinity to PD-L1 [89]. This unique affinity profile allows preferential binding to PD-L1 of tumor cells, suppressing the CD47 signaling pathway [89]. 6MW3211 demonstrates potent therapeutic efficacy in diverse mouse models and shows promising pharmacokinetics and safety profiles in vivo [89]. The coexpression of CD47 and PD-L1 on various human tumors, confirmed by multiplex fluorescent immunohistochemistry staining, supports the potential of 6MW3211 for clinical trials targeting PD-L1^+^ CD47^+^ cancers [89].

Besides, Chen et al. constructed an affinity-tuned anti-CD47 × PD-L1 BsAb (hBisAb) to improve antibody selectivity and therapeutic efficacy [90]. hBisAb was developed utilizing knobs-in-holes technology and a common light chain architecture for its IgG1 format. This humanized antibody demonstrates moderate affinity for CD47 and a highly potent affinity for PD-L1, as evidenced by kinetic rate constants obtained via surface plasmon resonance and cell-based assays. Specifically designed to prioritize PD-L1 binding, the antibody effectively blocks the PD-1/PD-L1 interaction and also inhibits the CD47/SIRPα axis [90]. This dual-action mechanism not only enhances T cell functionality but also significantly boosts phagocytosis of tumor cells by macrophages, outperforming monotherapies targeting either checkpoint alone [90]. In vitro and in vivo studies reveal that the hBisAb exhibits a remarkable selectivity for tumor cells over red blood cells, addressing a common challenge of CD47-targeted therapies by minimizing unwanted hematologic effects. This selectivity is further underscored by the antibody’s preferential binding to PD-L1-expressing cells in the TME, reducing off-target effects and improving therapeutic safety [90]. The bispecific antibody, particularly in its IgG1 form, has shown superior efficacy in promoting antibody-dependent cellular phagocytosis (ADCP) and DC-mediated T cell activation, leading to significant tumor growth inhibition and improved survival rates in syngeneic murine models. Notably, this approach mitigates the potential toxicity often associated with CD47 targeting, as evidenced by the maintenance of normal red blood cells counts and body weight in treated mice, highlighting the bispecific antibody’s enhanced antitumor efficacy and reduced side effects [90].

Furthermore, there are some other anti-CD47 × PD-L1 BsAbs have been reported. For instance, IBI322, was designed to improve therapeutic selectivity and efficacy by preferentially binding to PD-L1^+^CD47^+^ tumor cells, inducing tumor cell phagocytosis while minimizing impact on CD47^+^PD-L1^−^ cells like red blood cells [91]. Similarly, a dual-targeting fusion protein, IAB, effectively engaged both CD47 and PD-L1, demonstrating potent antitumor activity and playing a vital role in activating innate and adaptive immunity against tumors [92]. These innovative approaches underscore the potential of dual checkpoint blockade, simultaneously targeting CD47 and PD-L1, to improve therapeutic outcomes while mitigating toxicities associated with traditional antibodies.

## Anti-VEGF/PD-1 and anti-VEGF/PD-L1 BsAb

VEGF, induced by the hypoxic TME, stimulates endothelial cell proliferation and angiogenesis [93]. Additionally, VEGF exerts immunosuppressive effects, promoting the recruitment of immunosuppressive cells and hindering immune cell infiltration [94]. Combining anti-vascular targeting drugs with ICIs has demonstrated synergistic antitumor effects in various cancers, highlighting the potential of dual therapeutic strategies to address both angiogenesis and immune response in cancer treatment [95]. At the present stage, multiple anti-VEGF × PD-1 and anti-VEGF × PD-L1 BsAbs have been successfully developed for cancer immunotherapy.

Hassanzadeh et al*.* constructed a bivalent anti-PD-L1 × VEGF nanobody, which demonstrated efficient inhibition of angiogenesis in vitro [96]. Besides, the BsAb HB0025, targeting PD-L1 and VEGF, was developed using mAb-Trap technology. The preclinical studies showed that HB0025 was more effective in suppressing tumor growth compared to anti-PD-L1 antibody or VEGFR1D2 fusion protein alone [97]. Moreover, Xiong et al*.* developed a fully human bispecific single-chain diabody (BsDb) that targets VEGF165 and PD-1. This BsDb demonstrated high specificity, inhibiting VEGF165-induced activities in human umbilical vein endothelial cells and enhancing T cell proliferation and IFN-γ production [98]. In mouse models, the BsDb exhibited potent antitumor activity by suppressing angiogenesis and activating immune responses, suggesting its potential as a dual-targeting BsAb for cancer therapy [98]. Importantly, the phase 2 clinical trial assessed the efficacy and safety of AK112, a humanized IgG1 anti-VEGF × PD-1 BsAb, in combination with chemotherapy in advanced NSCLC [99]. The study included three cohorts with different treatment histories and genomic alterations. The confirmed objective response rates (ORR) in cohorts 1, 2, and 3 were 53.5%, 68.4%, and 40.0%, respectively [99]. The findings suggest that AK112 plus platinum-doublet presents promising antitumor activity and safety, providing a potential new treatment option for advanced NSCLC patients [99].

## Anti-4-1BB × PD-L1 BsAb

4-1BB (CD137) is an inducible costimulatory molecule expressed by activated NK and T cells [100, 101]. 4-1BB signaling, triggered by interaction with its ligand on professional antigen-presenting cells (APCs), activates pathways leading to enhanced cytokine generation, survival, proliferation, and immunological memory [102, 103]. In the TME, 4-1BB serves as a marker for tumor-specific cytotoxic T lymphocytes (CTLs) and is often co-expressed with PD-1 [104]. 4-1BB activation has shown promising antitumor responses in preclinical models, and the combination of 4-1BB agonist antibodies with PD-1/PD-L1 inhibitors synergistically enhances antitumor immunity [105–109]. Currently, the use of 4-1BB agonists combined with anti-PD-1 therapies faces a significant challenge especially systemic toxicity. For example, the clinical development of a therapeutic CD137 agonist antibody was discontinued due to dose-dependent hepatitis caused by the systemic activation of the 4-1BB pathway. Theoretically, the BsAb technique holds promise, as it could potentially activate 4-1BB through PD-L1 engagement, thereby enhancing tumor-specific T cell responses. This approach appears promising because PD-1 and 4-1BB are both co-expressed on tumor-specific CD8 + CTLs [110].

Several anti-4-1BB × PD-L1 BsAbs have been developed to enhance the therapeutic efficacy of ICIs by combining 4-1BB agonists with these inhibitors. MCLA-145 was engineered as an IgG1 molecule with specific modifications to the Fc CH3 domain to encourage heavy chain heterodimerization and to the CH2 domain to prevent Fc receptor binding. In vitro experiments indicated that MCLA-145 could potently activate T cells, strengthens T cell priming, differentiation, and immune memory, and exhibits superior antitumor activity compared to ICI comparators [110]. Importantly, MCLA-145 demonstrates no graft-versus-host disease and minimal adverse effects in non-human primates [110]. Mechanically, MCLA-145 functions by binding to PD-L1 on tumor cells and CD137 on T effector cells, facilitating the creation of an "immunological synapse." In this synapse, T cells can exposure to enhanced TCR signaling as PD-1 inhibition is relieved, and CD137 activation is intensified. Subsequent investigations have confirmed that the activation of CD137 signaling by MCLA-145 is conditional and occurs when neighboring cells express more than 5000 copies of PD-L1. This conditional activation offers potential advantages in safety and effectiveness. It is important to note that even under conditions of maximum saturation, MCLA-145 cannot trigger CD137 signaling in the absence of neighboring cells expressing PD-L1 [110]. Another bispecific antibody, ABL503, selectively activates 4-1BB signaling only in the context of PD-L1, avoiding dose-dependent toxicity observed in patients treated with anti-4-1BB agonistic antibodies [111]. ABL503 exhibits potent antitumor activity and improved safety profiles in preclinical models [111].

PRS-344/S095012 is developed to block the PD-1/PD-L1 pathway and localize 4-1BB co-stimulation to a PD-L1^+^ TME [112]. This bispecific molecule effectively combines ICI with TME-localized 4-1BB-mediated immunostimulation, demonstrating superior T-cell stimulation and antitumor activity in murine models compared to the combination of monoclonal antibodies [112]. Additionally, HK010, an Fc-mutated IgG4 anti-4-1BB × PD-L1 BsAb, exhibits a strong antitumor effect by simultaneously blocking PD-1/PD-L1 signaling and stimulating 4-1BB signaling [113]. HK010 shows potent antitumor immunity, induces durable antigen-specific immune memory, and is well-tolerated in preclinical models, suggesting a promising option for cancer immunotherapy [114]. Additionally, PM1003, a single-domain antibody towards a unique epitope of 4-1BB, is used in the engineering of multi-specific antibodies, such as anti-PD-L1 × 4-1BB BsAbs, to localize 4-1BB activation within the TME, resulting in potent inhibition of PD-L1 activity and antitumor activity with minimal toxicity in vivo [115].

Notably, in the phase 1 clinical trial (NCT03917381), the potential of DuoBody-4-1BB × PD-L1 (GEN1046), a first-in-class bispecific immunotherapy agent, was investigated in patients with advanced refractory solid tumors [116]. In preclinical models, GEN1046 demonstrated superior effects on T-cell proliferation, cytokine generation, and cytotoxicity function compared to clinically approved anti-PD-1/PD-L1 agents [116]. The ongoing first-in-human study revealed manageable safety, pharmacodynamic immune effects consistent with its mechanism of action, and early clinical activity, with a disease control rate of 65.6% (40/61) observed in patients, including those resistant to prior anti-PD-1/PD-L1 immunotherapy [116]. GEN1046's encouraging preclinical and clinical results suggest its potential to fill a clinical gap in patients with immunotherapy-relapsed or refractory disease, positioning it as a promising candidate for combination therapy with other immunotherapy agents [116]. In summary, these developments collectively provide novel strategies for cancer immunotherapy with enhanced efficacy and safety.

## Anti-LAG-3 × PD-L1 BsAb

LAG-3, an identified transmembrane protein on activated T cells and NK cells, delivers inhibitory signals to suppress T cell proliferation [117–119]. Monoclonal antibodies blocking LAG-3 and MHC-II interaction are currently being assessed for their potential antitumor effects [120, 121]. However, the coexpression of LAG-3 and PD-1 in tumors implies their involvement in T-cell exhaustion [122]. The combined administration of anti-PD-1/PD-L1 and anti-LAG-3 antibodies displays a synergistic ability to inhibit tumor growth, as evidenced in phase 2/3 trials where the combination of relatlimab and nivolumab yielded significantly prolonged PFS compared to nivolumab alone [123–125]. At present, the combination of relatlimab and nivolumab has been approved for advanced melanoma [123, 124]. Besides, combinations like ieramilimab (an anti-LAG-3 antibody) alongside spartalizumab (an anti-PD-1 antibody) have demonstrated sustained positive responses in various patient groups, including those with non-small cell lung cancer (NSCLC), melanoma, renal cell carcinoma, mesothelioma, and triple-negative breast cancer (TNBC). These responses were observed in patients who had not previously received anti-PD-1/L1 treatments and in melanoma and renal cell carcinoma patients who had undergone prior anti-PD1/L1 therapy [126]. The rationale for developing anti-LAG-3/PD-L1 bispecific antibodies arises from the observed coexpression in tumors, indicating a potential role in T cell exhaustion.

IBI323 and ABL501 represent promising BsAbs targeting LAG-3 and PD-L1, aiming to overcome the limited effectiveness observed in anti-PD-1/PD-L1 treatments for advanced tumors. IBI323 not only maintained the blockade activities of its parental antibodies but also introduced a novel cell-bridging function [127]. This innovative mechanism translated into heightened immune stimulatory activity in mixed leukocyte reactions and more robust antitumor responses in humanized mouse models, correlated with an increase in tumor-specific T cells [127]. Similarly, ABL501 was constructed from an anti-LAG-3 IgG4 antibody linked to a PD-L1-targeting scFv through a (G4S)3 linker, featuring a strategic S224P amino acid substitution to enhance stability. It effectively binds to its targets without eliciting Fc-mediated effector functions like ADCC and CDC, focusing its action on checkpoint blockade. In vitro experiments showed that ABL501 efficiently targeted both LAG-3 and PD-L1 pathways, outperforming individual anti-LAG-3 and anti-PD-L1 antibodies in enhancing the activation of effector T cells [128]. ABL501 demonstrated compelling in vivo antitumor efficacy in humanized xenograft models, underscoring its potential clinical significance [128]. The examination of immune profiles in peripheral blood highlights a heightened presence of the LAG-3 + PD-1 + memory CD4 + T cell subset in relapsed cholangiocarcinoma patients who underwent chemotherapy [128]. Notably, this subset predicts increased responsiveness to ABL501, providing valuable support for its ongoing first-in-human trial (NCT05101109) [128]. Mechanically, ABL501 promoted DC maturation and capacity to prime T cells, leading to improved cross-presentation of antigens and more robust CD8 + T cell activation. Additionally, ABL501 directly increased CD8 + T cell cytotoxicity against tumors. Its efficacy hinges on simultaneous engagement of LAG-3 and PD-L1, facilitating effective T cell-tumor cell interactions. By acting as a T cell engager and promoting T cell activation while blocking inhibitory signals, ABL501 orchestrates a potent antitumor immune response [128].

Besides, the anti-LAG-3 × PD-L1 BsAb FS118 exhibits promising preclinical and clinical results, offering a novel approach for cancer immunotherapy [129]. In preclinical investigations, FS118 demonstrated simultaneous binding to LAG-3 and PD-L1 with high affinity, surpassing the antitumor activity of the combination of anti-LAG-3 and anti-PD-L1 antibodies [130]. Mechanistic studies in syngeneic tumor mouse models revealed significant tumor growth suppression with the surrogate mLAG-3 × PD-L1 antibody. Notably, the murine surrogate led to decreased LAG-3 abundance of T cells, while the combination of individual antibodies increased LAG-3 expression [130]. Moreover, binding of the surrogate mLAG-3/PD-L1 antibody resulted in the rapid shedding of mouse LAG-3 into the blood [130]. In clinical studies, a phase 1 trial (NCT03440437) demonstrated the safety and tolerability of FS118 in patients with advanced, anti-PD-1/PD-L1-resistant cancers [131]. FS118 showed a recommended phase 2 dose of 10 mg/kg weekly, sustained pharmacodynamic activity, and an overall disease control rate of 46.5%, particularly notable in patients with acquired resistance to PD-1/PD-L1-targeted therapy [131]. This study supports the continued investigation of FS118 for patients with refractory cancers, highlighting its potential as an effective dual PD-L1 × LAG-3 blockade strategy. Apart from solid tumors, LAG-3-targeting BsAbs also exhibited promising efficacy in hematological malignancies. In the phase 1 clinical trial NCT03219268, the efficacy of anti-LAG-3 × PD-1 BsAb Tebotelimab was explored in patients with solid tumors or hematologic malignancies [132]. Notably, 34% of patients showed tumor reduction, with positive responses in various cancer types, including cases resistant to anti-PD-1 treatment [132].

## Anti-PD-1/CTLA-4 BsAb

CTLA-4 and PD-1 are immune checkpoints that inhibit various T cell functions, and their activation leads to T cell functional inhibition through multiple mechanisms [133]. CTLA-4, when induced upon activation, competes with CD28-mediated activation and removes costimulatory ligands from APCs [134]. PD-1, when expressed by T lymphocytes, acts as an inhibitor reducing cytotoxicity and cytokine generation. Both checkpoints are co-opted by tumors for immune evasion. Antibodies blocking CTLA-4 or PD-1 have shown antitumor activity in preclinical and clinical settings, and combination therapy has demonstrated improved responses in various cancers [135]. Besides, the TME analysis reveals a higher ratio of PD-1^+^CTLA-4^+^ cells in tumors compared to normal tissues, supporting the rationale for targeting PD-1^+^ CTLA-4^+^ cells to selectively block checkpoints in the TME while avoiding influences on normal tissues [136].

The systemic blockade of the PD-1/PD-L1 axis is foundational in cancer immunotherapy, especially considering its clinical significance and well-regarded safety profile. Therefore, a superior combination therapeutic should ensure it retains the effectiveness of PD-1 blockade without diminishing its capacity to interrupt PD-1 interactions with its ligands. However, the systemic CTLA-4 inhibition presents a higher risk of adverse effects. To mitigate these risks, anti-PD-1 × CTLA-4 BsAb provides refining the CTLA-4 inhibitory function to specifically target cells that co-express PD-1 and CTLA-4 within the TME [136]. For instance, MGD019 represents an advanced BsAb engineered to simultaneously target PD-1 and CTLA-4. This BsAb is uniquely designed with a tetravalent structure, utilizing a high-affinity anti-PD-1 monoclonal antibody, alongside an anti-CTLA-4 monoclonal antibody with properties that block ligands in a manner similar to the well-known ipilimumab [136]. The construction of MGD019 on the Dual-Affinity Re-Targeting platform with the 2 × 2 symmetric format, incorporating a hinge-stabilized IgG4 backbone. The unique structure of MGD019 allows for a robust blockade of PD-1 and a conditional inhibition of CTLA-4, tailored to the TME [136]. It mimics the in vitro PD-1 blockade efficacy of its anti-PD-1 precursor while modulating the CTLA-4 blockade to be most effective in cells expressing both PD-1 and CTLA-4. This specificity ensures localized CTLA-4 inhibition in the TME, enhancing safety by avoiding widespread Treg depletion [136]. Notably, the capacity of MGD019 capacity to block the interaction between PD-1 and its ligands with high efficiency, combined with its adaptable CTLA-4 blockade strategy, demonstrates significant antitumor activity with a manageable safety profile in patients with advanced solid tumors [136].

Moreover, the IgG4 Fc region of MGD019 confers a reduced capacity for Fc-mediated ADCC, thereby decreasing the inadvertent elimination of activated T cells and Tregs [136]. The effect of Treg depletion on the therapeutic efficacy and safety of ipilimumab remains under examination, yet the potential of Fc regions in anti-CTLA-4 antibodies to induce such depletion has been linked to both beneficial and detrimental outcomes in preclinical studies [137, 138]. By circumventing the depletion of Tregs while maintaining effective CTLA-4 blockade in the TME, MGD019 is designed to improve patient safety and uphold the beneficial effects of CTLA-4 antagonism. The immunosuppressive role of Tregs predominantly involves CTLA-4-mediated T cell exhaustion. Therefore, the blockade of CTLA-4 by MGD019 in the TME is expected to be sufficiently intense to compensate for the absence of Treg depletion [136]. This strategy, which prevents Treg reduction and ensures strong CTLA-4 inhibition in the TME, seeks to optimize the pivotal functions of these immune checkpoints in cancer immunotherapy, potentially yielding enhanced therapeutic benefits while minimizing adverse effects relative to conventional antibody treatments [136].

Additionally, the anti-PD-1/CTLA-4 BsAb QL1706 showed a manageable safety profile in a phase 1/1b study for advanced solid tumors refractory to standard therapies [139]. Across all patients at the recommended dose, the objective response rate was 16.9%, with a median duration of response of 11.7 months [139]. Notably, immunotherapy-naïve patients, particularly those with NSCLC, nasopharyngeal carcinoma, and cervical cancer, exhibited promising antitumor activities, with response rates of 24.2%, 38.7%, and 28.3%, respectively [139]. QL1706 is currently under evaluation in randomized phase 2/3 trials for further assessment of its efficacy [139]. Furthermore, MEDI5752, a novel monovalent bispecific antibody, enhances PD-1 blockade by selectively inhibiting CTLA4 on PD-1 + activated T cells [140]. It reduces the required dose for IL-2 secretion and rapidly internalizes and degrades PD-1. With a preference for tumor localization, MEDI5752 demonstrates superior in vivo activity compared to anti-PD-1 and anti-CTLA4 antibody combinations [140]. Two patients with advanced solid tumors showed robust partial responses to MEDI5752 treatment. This represents a significant advancement in cancer immunotherapy, offering distinct benefits by selectively targeting CTLA4 on PD-1 + T cells [140].

Cadonilimab is a tetravalent bispecific IgG1 antibody with an innovative Fc-null design, aming to eliminate Fc-mediated effector function for safety and efficacy considerations [141]. It exhibits biological activity comparable to the combination of CTLA-4 and PD-1 antibodies. Remarkably, cadonilimab displays higher binding avidity in a high-density PD-1 and CTLA-4 setting, offering potential advantages in tumor-like environments [141]. In the phase 1b/2 trial NCT03852251, the efficacy of cadonilimab was explored in advanced solid tumors [142]. Cadonilimab demonstrated encouraging efficacy, with objective response rates of 32.3% in cervical cancer, 18.2% in esophageal squamous cell carcinoma, and 16.7% in hepatocellular carcinoma [142]. These findings underscore the potential effectiveness of cadonilimab in achieving positive tumor responses in diverse advanced solid tumor types [142]. At present, Cadonilimab has received approval in China for treating relapsed or metastatic cervical cancer after platinum-based chemotherapy [143].

## Other BsAbs targeting PD-1/PD-L1 signaling

Besides the agents mentioned above, other BsAbs have been developed and are undergoing evaluation in clinical trials, such as anti-PD-L1/CD3, anti-PD-1/PD-L1, and anti-TIM-3/PD-L1 BsAbs. For instance, recent advances in the development of anti-PD-L1/CD3 BsAb have addressed key challenges associated with existing T-cell engagers. One notable innovation is the Protease-Activated PSTAGylated BiTE (PAPB), which is designed for solid tumors. PAPB incorporates a shielding polypeptide domain (PSTAG), a protease-activated linker, and a BiTE core with scFvs targeting PD-L1 and CD3. PAPB demonstrates a dose-dependent binding of the BiTE core to PD-L1 and CD3, with the ability to release the core in response to MMP2 in the TME, significantly prolonging its plasma half-life [144]. Furthermore, a novel anti-PD-L1/CD3 nanobody-based BiTE demonstrates cytotoxic activity on melanoma cells correlated with PD-L1 expression levels, highlighting its potential in treating PD-L1-overexpressing melanoma. Collectively, these advancements signify promising steps toward enhancing the safety, duration of action, and efficacy of anti-PD-L1/CD3 antibodies in the realm of solid tumor immunotherapy [145].

TIM-3 serves a critical role in cancer immunology as a negative regulator of immune response [146, 147]. In cancer, TIM-3 expression specifically identifies the most dysfunctional subset of CD8^+^ T cells, indicating their exhaustion [148]. Studies in preclinical cancer models demonstrate significant efficacy in co-blockading the TIM-3 and PD-1 pathways, both in solid and hematologic malignancies [149]. Ongoing clinical trials, particularly in solid tumors, are exploring the potential of anti-TIM-3 in combination with anti-PD-1, showcasing its promise as a target for cancer immunotherapy [52, 150, 151]. In the phase 1 study NCT03752177, LY3415244, a TIM-3/PD-L1 BsAb, was evaluated for safety and efficacy in patients with advanced solid tumors [152]. While some patients showed promising outcomes, such as a near partial response in a PD-1 refractory NSCLC patient (-29.6%), the trial faced challenges [152]. Notably, 16.7% of patients experienced clinically significant anaphylactic infusion-related reactions. All patients developed treatment-emergent antidrug antibodies (TE-ADA), impacting soluble TIM-3 target engagement and leading to early termination of the study [152]. Despite these challenges, the patient outcomes, particularly in the context of PD-1 refractory cancer, highlight the potential clinical impact of LY3415244, warranting further exploration and consideration in future studies.

## Conclusion and perspective

The advent and clinical validation of BsAbs targeting the PD-1/PD-L1 axis alongside other immune regulatory molecules mark a pivotal evolution in the landscape of cancer immunotherapy. This review has delved into the innovative strides made in the realm of BsAbs, particularly focusing on their development, mechanisms of action, and therapeutic potential in managing solid tumors. The exploration of BsAbs such as anti-TGFβ × PD-L1, anti-CD47 × PD-L1, and others, underlines a strategic endeavor to amplify antitumor immunity, overcome immune evasion, and address the limitations inherent in monotherapy approaches. While the therapeutic promise of these agents is underscored by both preclinical and emerging clinical successes, the journey towards their optimal integration into cancer care will require careful research and attention to detail.

Firstly, future research must concentrate on enhancing the specificity, efficacy, and safety profiles of BsAbs. This includes the development of next-generation BsAbs with reduced immunogenicity, improved tumor penetration, and tailored pharmacokinetics characteristics. Advanced molecular engineering techniques can facilitate the design of BsAbs that selectively accumulate within the TME, minimizing systemic exposure and associated toxicities. Besides, the complexity of the TME remains a formidable challenge to the efficacy of immunotherapies. Novel BsAbs that can modulate the suppressive TME, enhance antigen presentation, and promote T cell infiltration and activation within tumors are of particular interest. Strategies combining BsAbs with agents that disrupt physical barriers within the TME or neutralize suppressive cell populations could yield synergistic antitumor effects. Moreover, identifying predictive biomarkers for responsiveness to BsAb therapies is crucial. Comprehensive genomic, proteomic, and immunological profiling of tumors could unveil biomarkers that predict therapeutic response, guide patient selection, and facilitate personalized treatment approaches. This precision medicine approach would optimize therapeutic outcomes and mitigate the risk of adverse effects. Furthermore, the integration of BsAbs with other treatment modalities, including chemotherapy, targeted therapy, radiation, and other immunotherapies, holds great promises. Rational combination strategies based on mechanistic rationales and preclinical evidence can potentiate antitumor efficacy, counteract resistance mechanisms, and broaden the therapeutic window of BsAbs.

The exploration of BsAbs in cancer immunotherapy opens a new frontier in our fight against cancer, promising to enhance the precision, potency, and persistence of immune-mediated antitumor responses. The future of cancer treatment with BsAbs beckons a paradigm where the synergy of targeting multiple immune checkpoints or combining immune modulation with other therapeutic strategies can provide durable, effective, and safer treatment options for patients worldwide. The substantive prospects for BsAbs in cancer care not only highlight a promising therapeutic avenue but also underscore our collective commitment to turning the tide against cancer through immunological means.

## Data Availability

No datasets were generated or analysed during the current study.

## Abbreviations

PD-1: Programmed cell death 1
ITIM: Immune receptor tyrosine-based inhibitory motif
ITSM: Immune receptor tyrosine-based switch motif
BsAb: Bispecific antibody
TAA: Tumor-associated antigen
BiTE: Bispecific T cell engager
NSCLC: Non-small cell lung cancer
TGFβ: Transforming growth factor-β
DC: Dendritic cell
Treg: Regulatory T cell
TME: Tumor microenvironment
MDSC: Myeloid-derived suppressor cell
TIL: Tumor-infiltrating lymphocyte
TNBC: Triple-negative breast cancer
STING: Stimulator of an interferon gene
KIH: Knobs-into-hole
VEGFR1D2: Vascular endothelial growth factor receptor 1 domain 2
BsDb: Bispecific single-chain diabody
ORR: Objective response rate
APC: Antigen-presenting cell
CTL: Cytotoxic T lymphocyte
